# Effects of Deep Cryogenic Treatment on the Microstructure and Properties of Rolled Cu Foil

**DOI:** 10.3390/ma14195498

**Published:** 2021-09-23

**Authors:** Zhichao Dong, Xiangyu Fei, Benkui Gong, Xinyu Zhao, Jiwei Nie

**Affiliations:** School of Materials Science and Engineering, Shandong University of Technology, Zibo 255049, China; ZCDOng@sdut.edu.cn (Z.D.); zhaoxinyu@sdut.edu.cn (X.Z.); niejiwei789@163.com (J.N.)

**Keywords:** rolled Cu foil, deep cryogenic treatment, microstructure, mechanical properties, corrosion resistance

## Abstract

The development of fifth-generation (5G) communication and wearable electronics generates higher requirements for the mechanical properties of copper foil. Higher mechanical properties and lower resistance are required for flexible copper-clad laminate and high-frequency and high-speed Cu foil. Deep cryogenic treatment (DCT), as a post-treatment method, has many advantages, such as low cost and ease of operation. However, less attention has been paid to the impact of DCT on rolled Cu foil. In this study, the effects of DCT on the microstructure and mechanical properties of rolled Cu foil were investigated. The results show that as the treatment time increased, the tensile strength and hardness first increased and then decreased, reaching a peak value of 394.06 MPa and 1.47 GPa at 12 h. The mechanical property improvement of rolled Cu foil was due to the grain refinement and the increase of dislocation density. The dislocation density of rolled Cu foil after a DCT time of 12 h was determined to have a peak value of 4.3798 × 10^15^ m^−2^. The dislocation density increased by 19% and the grain size decreased by 12% after 12 h DCT.

## 1. Introduction

Rolled copper (Cu) foil is widely used in various fields such as communication [1], energy [2,3], and transportation [4] due to its excellent conductivity, ductility, electromagnetic shielding, and corrosion resistance. In addition, it has been used as a main component in copper-clad laminate (CCL) and lithium-ion batteries (LIBs) [5,6,7]. Advanced mechanical properties of Cu foil are required to adapt to the development of 5G communication [8], wearable electronics [9], and new energy vehicles (NEV) [10,11]. To date, various methods, such as ultrasonic surface rolling processing (USRP) [12], ultrasonic peening technique [13], and electrophoretic deposition (EPD) [14], have been applied to enhance the conductivity and corrosion resistance of Cu foil. However, the costly equipment and complex operations involved in these methods limit their practical applications.

Deep cryogenic treatment (DCT) refers to a process in which materials are treated at a temperature below −130 °C using liquid nitrogen (LN2) as a refrigerant. The DCT technique has been extensively used for ferrous and non-ferrous metals due to its ease of operation and low cost. DCT can refine the microstructure and inner stress of materials, which improves the mechanical behavior of the treated materials [15,16]. Liu et al. [17] investigated the mechanical properties and organizational structure evolution of a Cu plate after depositing Cu-rich Cu-Al-Si alloy using the cold metal transfer (CMT) technique. In addition, DCT can refine the size of grains and randomize the texture to improve the microhardness and tensile strength. Jovičević-Klug et al. [18] explored the effects of DCT on the surface chemistry and phase transformation of high-speed steels. Compared to the conventional heat treatment (CHT), DCT positively impacted the microstructure, making martensitic laths become sharper, smaller, and more oriented. Despite this progress, the effects of DCT on the surface chemistry of treated materials are still unclear. Dong et al. [19] found that the dislocation density of Al-Cu-Mn alloy significantly increased after DCT. Moreover, the tensile and yield strengths and elongations were promoted because of the grain refinement. Funk et al. [20] reported that DCT promotes the transformation of retained austenite while, at the same time, decreased electrical resistivity, Young’s modulus, and yield limit were observed due to the transformation of retained austenite. Li et al. [21] found that the rolling process at cryogenic temperature could induce the unique transformation of the microstructure. The ultra-low temperature restrains the atomic motion and dislocation slip, causing the kinking of grains and mechanical twinning. Lv et al. [22] investigated the effects of DCT on the mechanical behavior of bulk metallic glass. The samples exhibited a lower pop-in force and hardness after DCT because the densifiction of shear bands was promoted [23]. Yuan et al. [24] studied the drawability of aluminum alloy sheets treated by DCT at different temperatures. The deep drawability was improved as the temperature decreased. The cup drawn at ultra-low temperatures exhibited uniform thickness distribution and significant drawing height and load. Barylski et al. [25] investigated the effects of DCT on the wear and mechanical properties of Mg-Y-Nd-Zr alloy. It was found that the reduction of defects (dislocations and stacking faults) caused by DCT jeopardized the mechanical properties of the alloy, which can be improved by the aging process. Although extensive investigations of DCT have been conducted for many metals, there are fewer reports of DCT in the field of ultra-thin Cu foil. So, it is crucial to analyze the effects of DCT on Cu foil.

In this paper, the effects of DCT on the microstructure, mechanical, physical, and chemical properties of rolled Cu foil are studied. The results show that DCT significantly improved the tensile strength and nano-hardness of rolled Cu foil. The mechanism of mechanical properties improvement is proposed by investigating the microstructure, corrosion resistance, and dislocation density.

## 2. Materials and Methods

In this study, 12 μm Cu foils (Shandong Tianhe Rolled Copper Foil Co., Ltd., Heze, China, Ag < 100 ppm) were processed by DCT in LN2 for different times. Specifically, the Cu foils were soaked in LN2 at 88 K for 0 h, 12 h, 24 h, and 48 h, which corresponds to DCTCu-0, DCTCu-12, DCTCu-24, and DCTCu-48, respectively. 

The morphology and crystal orientation of Cu foil were investigated using a field emission scanning electron microscope (FE-SEM Apero S, FEI, Waltham, MA, USA) equipped with an electron backscatter diffraction (EBSD) system. The samples were processed by the hybrid ion milling system (IM4000, Hitachi, Tokyo, Japan) for surface observation and EBSD analysis. The applied parameters for ion milling are shown in Table 1. 

The crystal lattice of Cu foils was characterized using a high-resolution transmission electron microscope (HRTEM, Tecnai G2F 20, FEI, Waltham, MA, USA). The Cu foils were punched into 3 mm diameter foils. Subsequently, the thin TEM foils were thinned by ion milling using an argon ion mill (Gatan Pips II 695, Gatan, Pleasanton, CA, USA) at 4 kV.

The uniaxial tensile performance was evaluated by a universal machine (5967-E2, Instron, MA, USA) at room temperature with a crosshead speed of 1.0 mm min^−1^. The axial displacement was measured by an auto-extensometer (AutoX750, Instron, MA, USA). Each sample was measured three times.

The load-displacement curves, nano-hardness, and elastic modulus were characterized by a nano-indentation machine (Nanotest Vantage Alpha, Micro Materials, Wrexham, UK), with a standard Berkovich diamond indenter indentation depth of 500 nm.

The corrosion resistance was quantified by the Tafel test and A.C. impedance methods. The three-electrode system was used to examine the corrosion of Cu foil in a solution of 3.5 wt. %. sodium chloride (NaCl), which was degassed by purging with nitrogen for 20 min. The Cu foil was used as the working electrode, the platinum (Pt) plate was applied as a counter electrode, and the saturated calomel electrode (SCE) was utilized as a reference electrode. The Tafel curves were obtained using an electrochemical workstation (CHI760E, CHI Instrument, Shanghai, China), with the initial potential at open circle potential (OCP) plus 0.6 V, the final potential at OCP minus 0.6 V, and the scan rate at 0.01 V s^−1^, and electrochemical impedance spectroscopy (EIS) in a frequency range from 0.01 Hz to 100 kHz at open circle potential.

The grain size and dislocation density of the Cu foil were determined using X-ray diffraction (D8 Advance, Bruker AXS, Karlsruhe, Germany) with Cu Kα radiation (wavelength λ = 1.54 Å) at 35 kV and 30 mA. The scanning range, rate, and step size were 30°–100°, 2°/min, and 0.02°, respectively.

## 3. Results and Discussion

### 3.1. Mechanical Properties 

**Figure 1:**
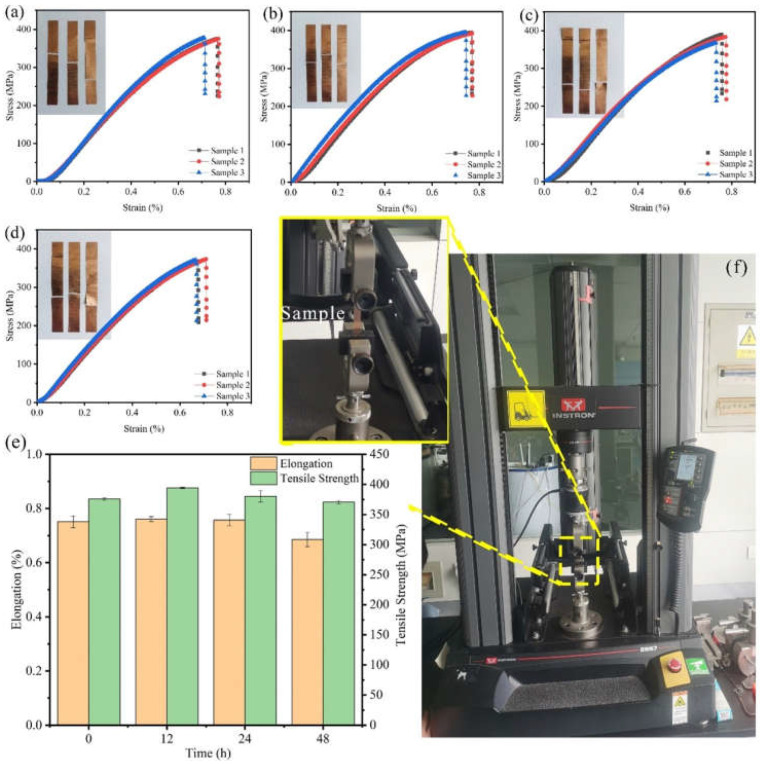
Stress–strain curves and images of Cu foils treated by DCT for (**a**) 0 h (**b**) 12 h, (**c**) 24 h, and (**d**) 48 h, (**e**) effects of DCT time on the uniaxial tensile performance, (**f**) a picture of the universal machine.

**Figure 2:**
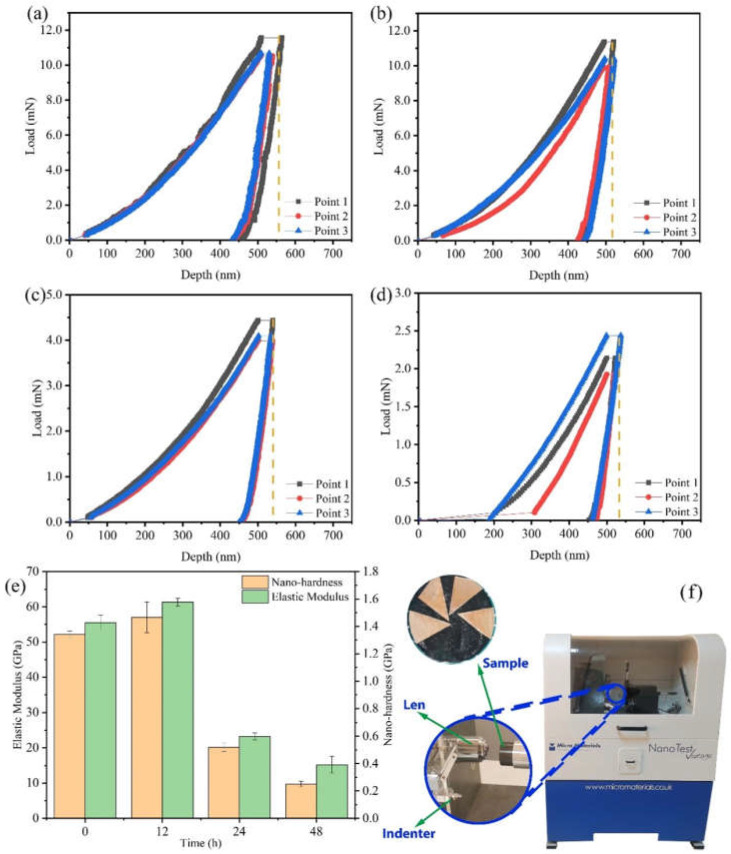
Load–displacement curve of Cu foil treated by DCT for (**a**) 0 h, (**b**) 12 h, (**c**) 24 h, (**d**) 48 h, and (**e**) the results of nano-hardness and elastic modulus on the nano-indentation; (**f**) a picture of the nano-indentation machine.

The mechanical properties of Cu foils processed by DCT for various times were characterized (Figure 1 and Figure 2). The stress–strain curves of Cu foils at DCT time, 0 h (DCT-0), 12 h (DCT-12), 24 h (DCT-24), and 48 h (DCT-48) are depicted in Figure 1a–d. The tensile strength and elongation first increased and then decreased with the increase in DCT time, peaking at 394.06 MPa and 0.761% at the DCT time of 12 h (Figure 1e). The real-time operation of the universal machine and nano-indentation during measurement are depicted in Figure 1f and Figure 2f, respectively. The nano-hardness of Cu foil samples after DCT for different times is shown in Figure 2. The hardness and elastic modulus reached the maximum value of 1.47 GPa and 61.34 GPa at the DCT time of 12 h. The nano-hardness of DCT-0, DCT-24, and DCT-24 were 1.340 GPa, 0.519 GPa, and 0.251 GPa, respectively. The uniaxial tensile strength and nano-hardness exhibited a similar trend under DCT, whereby they first increased then decreased with an increase in DCT time. 

### 3.2. Corrosion Resistance

**Figure 3:**
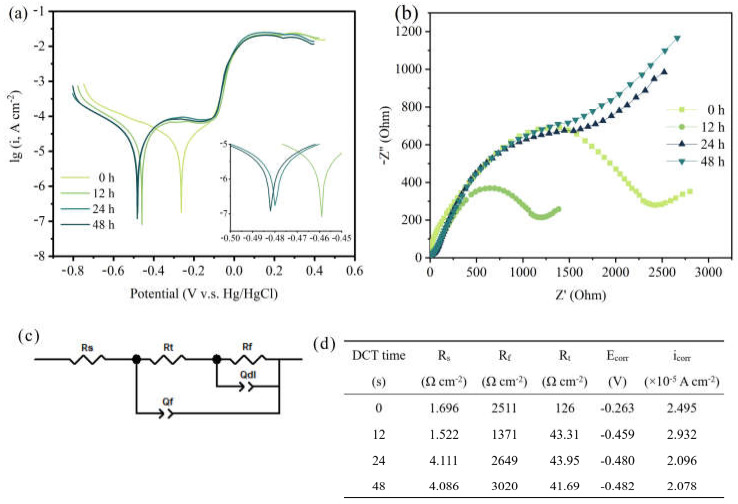
Corrosion resistance of (**a**) potentiodynamic polarization curves in the Tafel region, (**b**) the EIS curve, (**c**) the electrical equivalent circuit model, and (**d**) the corresponding data obtained from the equivalent circuit model.

To assess the corrosion resistance of the Cu foil for different DCT times, the electrochemical properties were tested (Figure 3). Figure 3a shows the potentiodynamic polarization curves. The linear-fitting results of the anode and cathode areas of the Cu foil at various DCT times were calculated (Figure 3d). The corrosion resistance measured by the Tafel extrapolation method exhibited a high self-corrosive current (2.932 × 10^−5^ A cm^−2^), which indicates that the sample of DCT-12 had poor corrosion resistance.

The EIS curve is depicted in Figure 3b. The ohmic resistance between the electrolyte and electrode is described by the intersection of the semicircle and the real axis in the high frequency region [26]. The charged electron transfer resistance is depicted by the slope of the line in the middle frequency region [27]. The curves illustrate that the DCT-12 had better electrochemical performance than the others. The former had lower charge transfer resistance and better conductivity [28], leading to a faster electron transfer speed. The EIS spectrum is also consistent with the results of the Tafel curve. The electrical equivalent circuit model and the corresponding data obtained from the equivalent circuit model are shown in Figure 3c,d. The R_s_ is the resistance of electrolyte. The R_t_ and R_f_ are the outer resistance of the electrochemical system and the resistance of the inner layer that covers the metal surface [29,30]. The passivation film formed on the Cu foil in the corrosive environment had better corrosion resistance at DCT-48 (3020 Ω cm^−2^). The corrosion resistance of Cu foils first increased then decreased with the increase in DCT time. 

### 3.3. XRD Analysis

**Figure 4:**
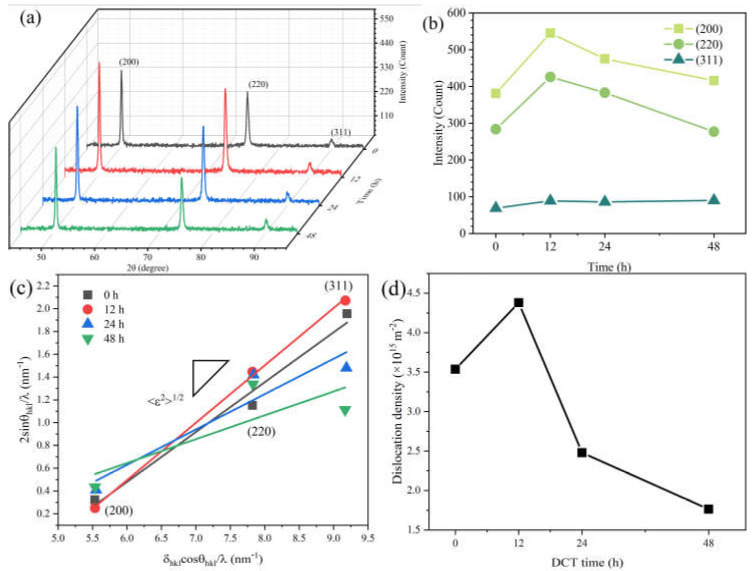
XRD patterns of Cu foil at various DCT times. (**a**,**b**) the intensities of different diffraction peaks, (**c**) the relationship between *2sinθ_hkl_/λ* and *δ_hkl_cosθ_hkl_/λ*, and (**d**) the result of dislocation density.

XRD patterns of samples at different DCT time are shown in Figure 4a. The diffraction peak intensity ratio of the Cu foil was different compared to the standard powder diffraction file (PDF) card. The main peak of the Cu foil was (200) (shown in Figure 4b), instead of (111) in the standard Cu PDF card due to the intense deformation during the rolling process. The intensities of different diffraction peaks are shown in Figure 4b. After a DCT process of 12 h, the intensity of (200) and (220) were enhanced while the intensity of (311) was unchanged. The lattice constants were also changed. This phenomenon is also observed in other literatures [31,32]. The lattice paraments of Cu foils were calculated to be 0.36117 nm, 0.36137 nm, 0.36175 nm, and 0.36135 nm after a DCT time of 0 h, 12 h, 24 h, and 48 h, respectively.

The XRD profiles show no peak shift of the Cu phase during the DCT process (Figure 4a). However, the full width at half maximum (FWHM) value obtained by the XRD spectrum broadened and then shrank with the increase in DCT time. The diffraction peaks (200), (220), and (311) were utilized for analysis. The average microstrain *<ε^2^*>^1/2^ was derived from the FWHM value of the XRD peaks using the following [33,34]:

(1) $$\delta_{e,\;hkl}=2{\langle \varepsilon^{2}\rangle }^{1/2}tan\theta_{hkl}$$

where *δ_e,hkl_* is the internal stress or lattice distortion, and *θ_hkl_* is the diffraction angle. The size-induced broadening *δ_D,hkl_* can be established by:

(2) $$\delta_{D,\;hkl}=\frac{\lambda }{Dcos\theta_{hkl}}$$

where *λ* is the wavelength (0.15405 nm), and *D* is the average grain size. *D* in the Cu foil samples fell in the microscale range, which is large enough to ignore the size effect. Hence, the line broadening (*δ_hkl_*) can be described as:

(3) $$\frac{\delta_{hkl}cos\theta_{hkl}}{\lambda }=2{\langle \varepsilon^{2}\rangle }^{1/2}\frac{sin\theta_{hkl}}{\lambda }$$

Meanwhile, *δ_hkl_* can be expressed by Equation (4),

(4) $$\delta_{hkl}=\sqrt{\delta_{hklm}^{2}-\delta_{hkl0}^{2}}$$

where *δ_hkml_* is the FWHM value of the (hkl) diffraction peak of Cu foil samples, and *δ_hkml_* is the FWHM value of the (hkl) diffraction peak of standard Cu [35].

The *<ε*^2^>^1/2^ can be obtained from Equation (3) from the linear relationship obtained between *δ_hkl_cosθ_hkl_/λ* and *2sinθ_hkl_/λ,* as shown in Figure 4c. The diffraction peaks of (200), (220), and (311) are used to simulate and obtain a slash. The slope of the slash is *<ε*^2^>^1/2^. The dislocation density can be described from the *<ε*^2^>^1/*2*^ by following [36,37]:

(5) $$\rho =\frac{3\sqrt{2\pi }{\langle \varepsilon^{2}\rangle }^{1/2}}{Db}$$

where *ρ* is the dislocation density, *D* is the grain size, and *b* is the Burgers vector, which can be calculated by Equation (6):

(6) $$b=\frac{a}{\sqrt{2}}$$

where *a* is the lattice parameter of Cu. The dislocation densities calculated by Equations (1)–(6) are shown in Figure 4d.

The dislocation densities significantly increased at a DCT time of 12 h and then decreased with a further increase in DCT time. The dislocation densities were calculated to be 3.536 × 10^15^ m^−2^, 4.3798 × 10^15^ m^−2^, 2.4797 × 10^15^ m^−2^, and 1.7640 × 10^15^ m^−2^, for DCT-0, DCT-12, DCT-24, and DCT-48 samples, respectively. 

The results of the XRD profile show that a long period of the DCT process can reduce the dislocation density, which is beneficial to release the distortion energy and improve the corrosion resistance of Cu foil. The tensile strength and hardness decrease as the dislocation density decreases. However, within a short period of DCT time (12 h in our experiment), the dislocation density increased significantly. The long-term low-temperature environment stabilizes the base tissue, releases the residual stress in the tissue, and partially misaligns as the structure disappears.

### 3.4. EBSD Analysis

**Figure 5:**
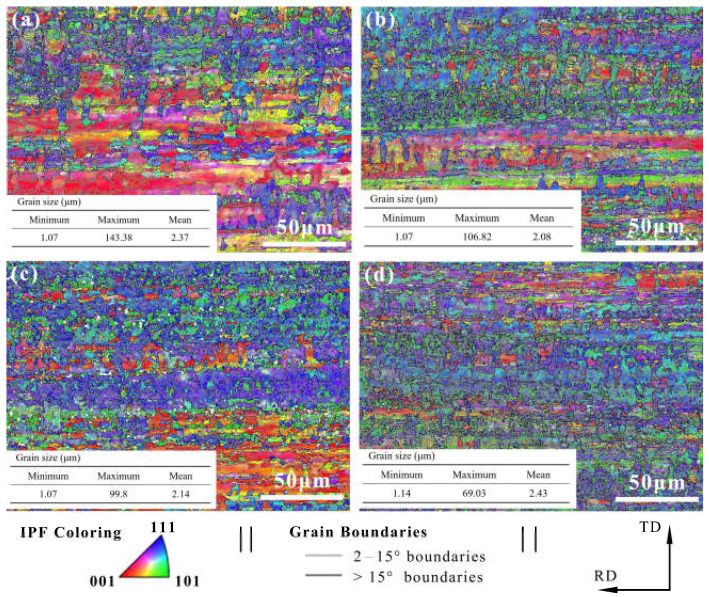
EBSD results at various DCT times of (**a**) 0 h, (**b**) 12 h, (**c**) 24 h, and (**d**) 24 h.

**Figure 6:**
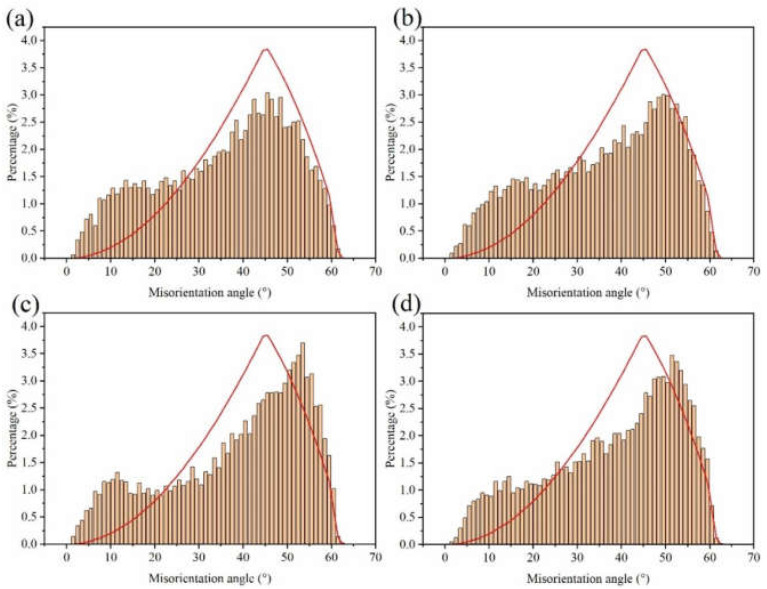
Distribution of the boundary misorientation angle at (**a**) DCT-0, (**b**) DCT-12, (**c**) DCT-24, and (**d**) DCT-48.

The electron backscatter diffraction (EBSD) measurements were used to show the grain size and morphology of our samples (Figure 5). The inverse pole figure (IPF) obtained by orientation imaging maps (OIM) was used to study the grain orientation. Furthermore, the grain boundary was used to evaluate the grain size and morphology. The Cu foil had an approximately smooth surface with horizontal stripes produced by the rolling deformation, the stripes being distributed along the rolling direction. The distribution of the misorientation angle in Figure 6 shows that the crystallite boundaries were mainly high angle boundaries in nature.

The grain orientation and the frequency of misorientation angle during the DCT process are shown in Table 2.

**Figure 7:**
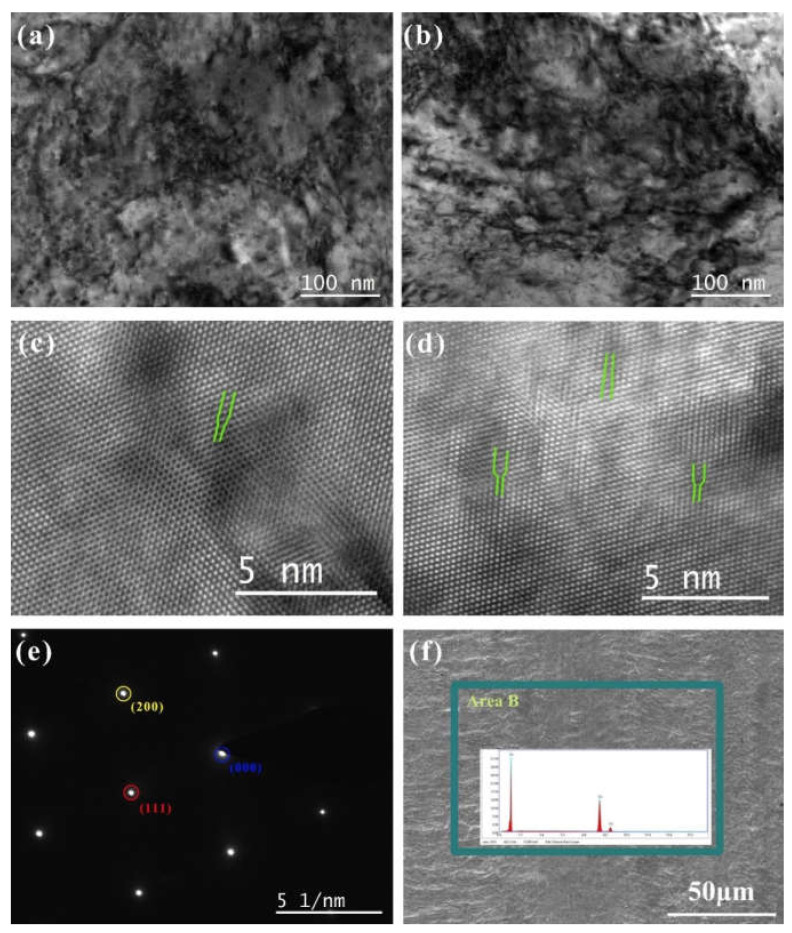
The morphology of TEM and HRTEM of Cu foil at DCT time (**a**,**c**) 0 h and (**b**,**d**) 12 h; (**e**) the SAED spectrum; (**f**) the SEM morphology of the original Cu foil and corresponding elements analysis.

The lattice plane of (111) gradually increased with the increase in DCT time and showed obvious (111) crystal orientation. Misorientation angles from 2° to 15° are defined as low angle grain boundaries (LAGBs), while angles >15° are defined as high angle grain boundaries (HAGBs). The LAGBs for Cu foils under different DCT times are 76.7%, 74.4%, 73.2%, and 69.8%, respectively. It can be seen that the frequency of LAGB decreased slowly due to the increase in DCT time, showing that the DCT promotes the transition from LAGBs to HAGBs. The fraction of LAGBs decreased because of the formation of extrinsic dislocation, as shown in Figure 7. LAGBs may have reacted with the extrinsic dislocation, leading to the transformation of LAGBs to HAGBs [38,39]. The Cu foils experienced thermal shrinkage and volume change when being dropped into the LN2 [32]. Due to shrinkage strain, the stored deformation energy acts as the driving force for the generation and mobility of dislocations, which eventually leads to dislocation interactions [17]. The grain size is depicted in Figure 5. The grain size first decreased then increased with the DCT time, reaching a minimum value of 2.08 μm at a DCT time of 12 h.

### 3.5. SEM/TEM Observation

The SEM morphology of the original Cu without DCT processing, and the elements analysis results, are shown in Figure 7f. The Cu foil had prominent horizontal stripes on its surface due to the rolling process. The element analysis demonstrated that the main component was Cu. The element Ag was not detected due to the very low concentration of Ag.

To observe the dislocation of the original Cu foil and the Cu foil treated by DCT, TEM, high-resolution electron microscopy (HRTEM), and selected-area electron diffraction (SAED) were performed (Figure 7a–e). The dislocation density of the DCT-0 was lower than that of the DCT-12. The sample in the environment of LN2 changed in volume as the temperature dropped to a very low temperature. The image of HRTEM shows the presence of the edge dislocation and that the DCT-12 has more edge dislocation in Figure 7c,d. The compression deformation force produced by the volume changes provided a driving force to promote the formation and migration of dislocation [32,40]. It significantly improved the mechanical performance, as shown in Figure 1 and Figure 2. However, the improvement of the dislocation density reduced the corrosion resistance of the Cu foil.

## 4. Conclusions

In this work, the effects of DCT time on the microstructure and properties of rolled Cu foil were investigated. The main conclusions are as follows:

- The DCT can improve the mechanical properties of Cu foil; the tensile strength and hardness reached a maximum value (394.06 MPa and 1.47 GPa, respectively) at a DCT time of 12 h. Corrosion resistance was negatively related to the mechanical properties.
- The grain orientation of (111) was improved with the increase in DCT time. Meanwhile, DCT increased the fraction of HAGBs and the dislocation density. The dislocation density of rolled Cu foil after a DCT time of 12 h increased by 19%, which was determined to have a peak value of 4.3798 × 10^15^ m^−2^.
- The tensile strength and hardness were related to the grain size and dislocation density. After DCT, LAGBs may react with the extrinsic dislocation, leading to the transformation of LAGBs to HAGBs.

## Figures and Tables

**Table 1:** The parameters for ion milling.

Parameter	Value
Discharge voltage/kV	1.5
Acceleration voltage/kV	4
Ion beam irradiation angle/°	80
Specimen rotation speed/r min^−1^	25
Ar gas flow/cm^3^ min^−1^	1

**Table 2:** The results of the recrystallized fraction and the grain orientation fraction.

DCT Time	Grain Boundary Fraction (%)		Grain Orientation Fraction (%)		
	2°–15°	>15°	(001)	(101)	(111)
0	76.7	23.3	38.5	31.5	30.0
12	74.4	25.6	16.5	41.0	42.5
24	73.2	26.8	26.8	29.1	44.1
48	69.8	30.2	10.8	38.7	50.5

## Data Availability

The data presented in this study are available on request from the corresponding author.
